# Emission energy, exciton dynamics and lasing properties of buckled CdS nanoribbons

**DOI:** 10.1038/srep26607

**Published:** 2016-05-23

**Authors:** Qi Wang, Liaoxin Sun, Jian Lu, Ming-Liang Ren, Tianning Zhang, Yan Huang, Xiaohao Zhou, Yan Sun, Bo Zhang, Changqing Chen, Xuechu Shen, Ritesh Agarwal, Wei Lu

**Affiliations:** 1 National Lab for Infrared Physics, Shanghai Institute of Technical Physics, Chinese Academy of Science, Shanghai, 200083, China; 2 Wuhan National Laboratory for Optoelectronics, Huazhong University of Science and Technology, Wuhan 430074, China; 3 Shanghai Advanced Research Institute, Chinese Academy of Science, Shanghai 201210, China; 4 Department of Materials Science and Engineering, University of Pennsylvania, Philadelphia, Pennsylvania, 19104, United States

## Abstract

We report the modulation of emission energy, exciton dynamics and lasing properties in a single buckled CdS nanoribbon (NR) by strain-engineering. Inspired by ordered structure fabrication on elastomeric polymer, we develop a new method to fabricate uniform buckled NRs supported on polydimethylsiloxane (PDMS). Wavy structure, of which compressive and tensile strain periodically varied along the CdS NR, leads to a position-dependent emission energy shift as large as 14 nm in photoluminescence (PL) mapping. Both micro-PL and micro-reflectance reveal the spectral characteristics of broad emission of buckled NR, which can be understood by the discrepancy of strain-induced energy shift of A- and B-exciton of CdS. Furthermore, the dynamics of excitons under tensile strain are also investigated; we find that the B-exciton have much shorter lifetime than that of redshifted A-exciton. In addition, we also present the lasing of buckled CdS NRs, in which the strain-dominated mode selection in multi-mode laser and negligible mode shifts in single-mode laser are clearly observed. Our results show that the strained NRs may serve as new functional optical elements for flexible light emitter or on-chip all-optical devices.

Strain-engineering has been demonstrated to be a very efficient way to tune the optical and electronic properties of materials, which may lead to new functional devices with great flexibility^1^,^2^,^3^,^4^,^5^,^6^,^7^,^8^,^9^. Especially in nanomaterials, high crystal quality and much larger elastic limit because of large surface-volume ratio, facilitate the strain-related new functionalities. Many remarkable reports on tuning electronic and optical properties of diverse nanostructures by strain-engineering have been published^8^,^9^,^10^,^11^,^12^,^13^,^14^,^15^,^16^,^17^. For example, strain effect on electron-transport of carbon nanotubes^1^,^8^; silicon nanomembranes and nanowires^10^,^11^,^12^,^13^,^14^; piezoelectricity or optical-mechanical coupling of ZnO, CdS nanowires^15^,^16^,^17^,^18^,^19^,^20^,^21^,^22^,^23^,^24^; strain-related bandgap-engineering and electronic properties in 2D nanomaterials^25^,^26^,^27^,^28^. And very recently, hundreds of meV exciton energy shift modulated by strain were observed in both GaAs and CdS nanowires^29^,^30^. Undoubtedly, Strain engineering provided a new method to tailor the bandgap of materials instead of traditional ways such as component ratio control, quantum confinement effect.

Nanoribbons (NRs) with an out-of-plane buckled geometry, which have great bendability and mechanical stretchability, are functional nanostructures with great application potential in both electronic and optical device. In last decade, both mechanical and electronic properties of these flexible NR devices have been intensively investigated^31^,^32^,^33^,^34^,^35^,^36^,^37^. For example, stretchable “wavy” silicon was functioned as flexible field-effect transistors^31^,^32^,^33^,^34^, the buckled GaAs NRs with very high stretchability and compressibility^36^, 70% enhancement of piezoelectric effect in wavy PZT ribbons^37^, *etc*. Some NRs (*e.g.* CdS NR, ZnO NR) can also serve as laser sources, waveguides and even semiconductor optical cryocooler, which are building blocks for all-optical on-chip devices^38^,^39^,^40^,^41^,^42^,^43^. It is highly desirable to realize flexible NR-based optical devices, but in above reports, NRs usually have a length of millimeter and perfect alignment along the prestrain direction of elastomeric substrate, which allows for a periodic buckling just via adhesive Van der Waals forces between NRs and elastomeric substrate. Neither of them is fulfilled (maximal hundred micrometers and not good alignment) for CdS or ZnO NRs, thus it is hard to get a periodic buckling with a stable geometry. Moreover, flexibility of NR-based optical devices inevitably induces deformation and thus strain effect on materials which is expected to modulate the optical properties of NR, just like buckled nanowires^29^. But as far as we know, the optical tuning (*e.g.* PL, exciton’s dynamics and lasing properties) of NRs by strain-engineering has rarely been investigated before^44^,^45^. In this work, CdS, of which nanostructures have been widely investigated in their optical properties^46^,^47^,^48^,^49^,^50^,^51^, is chosen as the test bed because of its high optical quality and very large fracture strength limit^30^. We present a new method to fabricate wavy structured CdS NRs and studied their optical response to strain. CdS NRs were wrinkled to a nearly uniform buckled structure, which was well characterized by atomic force microscope (AFM) and PL mapping (a periodic emission shift with 14 nm). Moreover, the tuning of emission energy, lifetime of excitons and lasing properties under strain were investigated by using micro-PL, micro-reflectance, and time-resolved PL techniques, respectively. This uniform buckled structures and related optical tuning may greatly expand their potential applications in large-scale integrated flexible light-emitting devices.

## Experiments and measurements

CdS NRs were grown via the vapor-liquid-solid method at the temperature of 850 °C, which have the wurtzite crystal structure with the [0001] direction (c-axis) perpendicular to the nanoribbon long axis (a-axis)^42^,^52^. To measure the optical properties of buckled NRs, we utilized our home-made micro-PL and micro-reflectance setup based on a microscope (as shown in Supplementary Materials, Figure S1). A semiconductor laser (457 nm continuous laser used for PL and 355 nm pulse laser used for lasing) was focused to a spot of ~1 μm by a 50X (NA, 0.5) objective. The emission was collected by the same objective and directed to spectrometer. Spatial scanning measurements were performed in cryostat mounted on a translation stage with the spatial resolution of 1 μm. All the spectral measurements were performed at a temperature of 77 K to ensure that A- and B-exciton of CdS are clearly visible in the spectra. The time-correlated single-photon-counting system (TCSPC) was adopted to study the exciton dynamics.

## Results and Discussions

**Figure 1:**
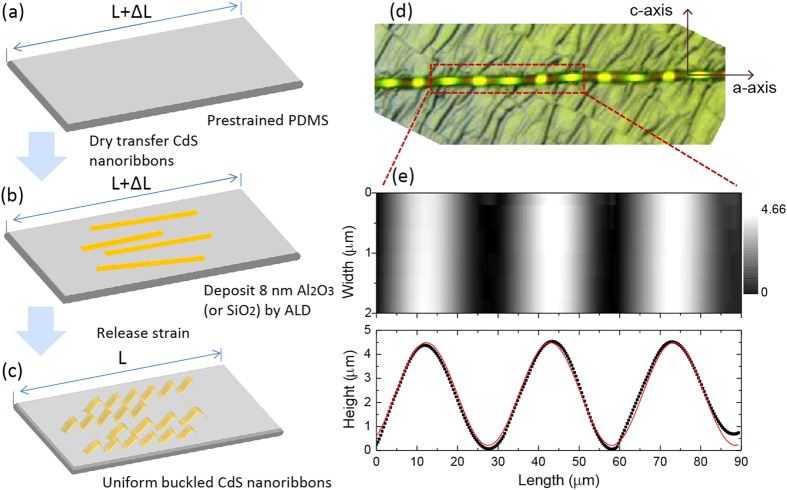
The fabrication process and the characteristics of the samples. (**a–c**) Schematic diagrams of the fabrication process for buckled CdS NRs. (**d**) Optical image of a typical buckled CdS NR. (**e**) Atomic Force Microscopy image (top panel) of the buckled CdS NR which is shown in (**d**), and the surface height (bottom panel) of CdS as a function of position along wavy CdS NR on PDMS, the red solid curve represent sinusoidal fits to the data.

To fabricate out-of-plane buckled CdS NRs, shown in Fig. 1(d), a prestrained PDMS substrate was prepared by a custom-built strain stage. We first dry transferred as-grown CdS NRs from the growth substrate to a polyimide film, and then carefully contacted the NRs on polyimide film onto the prestrained PDMS surface and mechanically slid it along the PDMS prestrain direction. In this manner, most NRs are transferred to PDMS with the long axis roughly following the prestrain direction. But unlike the buckled GaAs or PZT ribbons^36^,^37^, here, adhesive Van der Waals forces between NRs and PDMS cannot ensure a stable periodic buckled CdS NRs formed. To overcome this limit, we deposited a 5 nm thick Al_2_O_3_ film on the NRs by Atom Layer Deposition to tightly fixed NRs on PDMS surface without any influence on PL measurement. A comparison of PL spectra of nanoribbon with and without a layer of Al_2_O_3_ and a corresponding discussion are shown in Supplementary Materials (Figure S2). After that, we released the strain of PDMS and obtained CdS NRs with wavy geometry (Fig. 1(a–c)).

The optical image of a typical buckled NR is presented in Fig. 1(d). One can see clearly that the periodic out-of-plane buckled structure of a single CdS NR is formed. To understand its geometrical characteristics, AFM with tapping mode was adopted to scan along the buckled surface of NR and its atomic force micrograph mapping is shown in Fig. 1e (top panel). For clarity, the profile of buckled ribbon (black dots) is also shown at bottom panel; a sinusoidal profile of buckled CdS NR, which is the same as that of buckled silicon ribbons^36^, is clearly visible. The red curve is the sinusoidal fit to experimental data, from which the amplitude of 2.13 μm and a period of 30.5 ± 1.4 μm are obtained.

**Figure 2:**
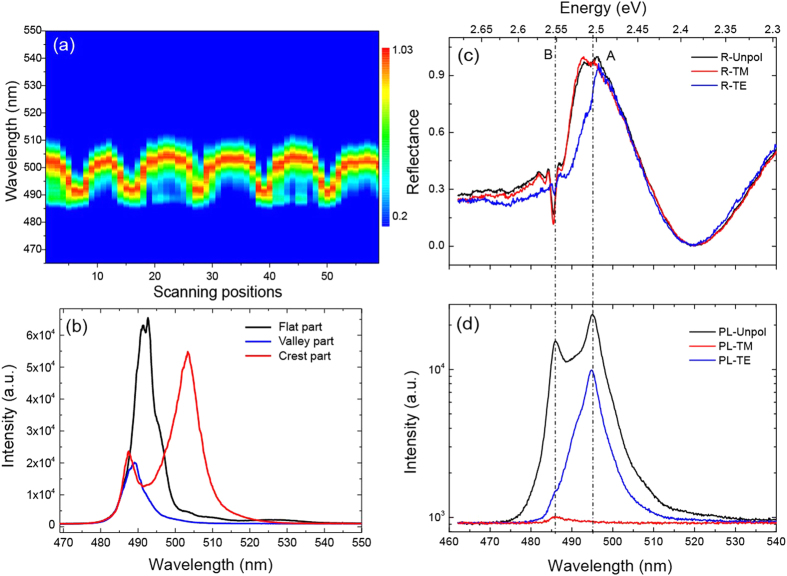
PL and Polarization-resolved reflectance spectra measured on periodic buckled CdS NRs. (**a**) Spatially resolved PL mapping of the buckled NR, exciton emission peak shows a periodic energy shift. (**b**) Spectra measured at flat part, valley part and crest part are presented, respectively. Polarization-resolved reflectance (**c**) and PL (**d**) spectra measured at the crest part of thin buckled CdS NR. (Here, to avoid the resonant modes of cavity, formed between the top and bottom surface of thick NR, in the reflectance spectra, a thin buckled NR (thickness, 200 nm) with small tensile strain was selected to perform micro-reflectance measurements). Features corresponding to the A- and B-exciton are clearly observed from both the reflectance and PL spectra.

Strain-induced exciton energy shifts have been demonstrated in bent ZnO, CdS and GaAs nanowires recently^19^,^29^,^30^. Out-of-plane CdS buckled NRs actually have a similar buckled geometry as bent nanowires, which the tensile strain localized at the outer surface and the compressive strain at the inner surface, respectively. Thus it is expected that the exciton energies of CdS should be modulated by the periodic strain variation of the buckled NR. Figure 2(a) shows the micro-PL spectral mapping, including around 60 spectra measured on the top surface of buckled nanoribbon with a scanning step of ~2.5 μm along the long axis. One can see that the emission peak shifts periodically, which can be attributed to the tensile and compressive strain alternation along the buckled NR. The waterfall plot shown in Supplementary Materials, Figure S3, shows that the maximum redshifted emission peaks (~503.5 nm) and the emission peaks (~489.5 nm) correspond exactly to the crest and the valley of the buckled NR, respectively. Due to the nearly perfect sinusoidal geometry, the redshifted emission peaks measured at six crests of the buckled NR are around 503.5 nm, which show uniform and periodic tuning of emission energy. As mentioned above, the redshift of the exciton emission is attributed to the tensile strain (*ε*_*top*_) localized at the top surface of the crest part, which can be roughly estimated by the local radius of curvature, *r*, and thickness, *d*, of NR (*ε*_*top*_* = d/2r*)^19^. As characterized by AFM, the NR has a thickness of ~0.35 μm, and the inscribed circle radius of the crest part is ~2.75 μm. With these values, the tensile strain *ε*_*top*_ can be calculated to be around 6.36%, resulting in the redshift of exciton emission of ~14 nm (~68 meV). This redshift amount may be further improved by increasing the pre-tensile strength of PDMS to obtain a larger curvature. For comparison, we also measured the PL spectrum from the flat part of NR on PDMS (as shown in Fig. 2(b), black curve). The red curve (measured at the crest part) is clearly redshifted and the blue curve (obtained from the valley region) shows a little bit blueshift. This is consistent with the previous results of exciton energy under tensile or compressive strain in CdS nanowires^29^. The redshifted emission peak shows broadening compared to the black spectrum, this can be attributed to the strain gradient distribution both along the thickness direction and along the a-axis of the NR. In addition, other possibility is that bending deformation-induced broadening of the gaps between different subbands in the conduction band of CdS, which was predicted by theoretical calculation^29^. At last, one should note that the strain-induced piezoelectric field in CdS NRs may affect the PL properties. In regard to this issue, some previous literatures have demonstrated that the influence of piezoelectric field on the PL is strongly dependent on the size of nanostructures and the binding energy of excitons^53^,^54^,^55^. For CdS NRs, it is believed that the PL redshift is dominated by the tensile strain-induced bandgap shift, and the piezoelectric field may have slight contribution for thin buckled CdS NRs (~200 nm)^54^.

Tensile strain induces redshift and broadening of emission peak, but beside that, the other emission peak at the wavelength of 489 nm is always accompanied the redshifted emission peak. This phenomenon is quite similar to the double peaks observed on bent CdS nanowires, in which the PL peak at high energy side was shown to be originating from the emission of B-exciton at inner part (compressive strain part) of a bent nanowire^29^. But in a buckled NR, we notice that laser mainly excite the tensile part because of penetration depth of laser is no more than 100 nm^56^,^57^, Even though 30% of the incident light may penetrate more into the CdS NRs and excite the compressive part, considering that the emission coming from the compressive part may be re-absorbed by the tensile part because of tensile strain-induced redshifted energy band, it is hard to generate a strong emission peak at around 489 nm, as shown in Fig. 2(b). Thus, this phenomenon is totally different from the in-plane bent nanowire where both tensile and compressive parts are covered by the laser spot, and the emission peak at 489 nm should not originate from the compressive part. To explore its fundamental properties and the physical mechanism, the micro-PL and micro-reflectance spectral techniques with different polarization configurations were used to study the excitons’ characteristics. To avoid the impacts from resonant modes of cavity, usually formed between the top and bottom surface of thick NR, in the reflectance spectra, a thin NR (thickness, 200 nm) was selected to perform micro-reflectance measurements. Figure 2(c,d) shows the micro-reflectance (c) and micro-PL (d) spectra with different polarization configurations, which are measured from the crest. One can see that the polarization measurements in reflectance show different spectral characteristics for the two emission peaks in PL spectra. As known, CdS has a wurtzite crystal structure, the conduction band is s-like state, whereas the valence band is p-like state, which is split into three bands (labeled as A, B, and C) due to the crystal-field effect and spin-orbit interaction^58^. According to the selection rules, all three excitonic transitions are allowed for transverse electric (TE) polarization (E-field component of light perpendicular to the c-axis of NR), while in transverse magnetic (TM) polarization (E-field component of light parallel to c-axis), the A-exciton is entirely forbidden, while B and C excitons are allowed. The redshifted emission peak (~2.505 eV) is originally TE polarized in both polarized reflectance and PL spectra, which allows us to assign it to A-exciton. Whereas for the emission peak at the high energy side (~2.551 eV), the polarized reflectance spectra show that both TE and TM polarizations are allowed, and thus it is reasonable to attribute this emission peak to B-exciton. Compared to the energy difference (~20 meV) between A- (2.540 eV) and B-exciton (2.560 eV) of unstrained CdS^29^, the tensile strain-induced the A- (2.505 eV) and B-exciton (2.551 eV) energy difference becomes larger (~46 meV) in the buckled CdS NR. This can be understood by the different energy shift trend of A- and B-exciton under uniaxial strain, which has also been experimentally shown in refs [29 and 30]. Experimental study of CdS nanowires show that the A-exciton shifts more than that of B-exciton under the same strain, which explains why we observed a larger energy splitting between A- and B-exciton of buckled NR.

**Figure 3:**
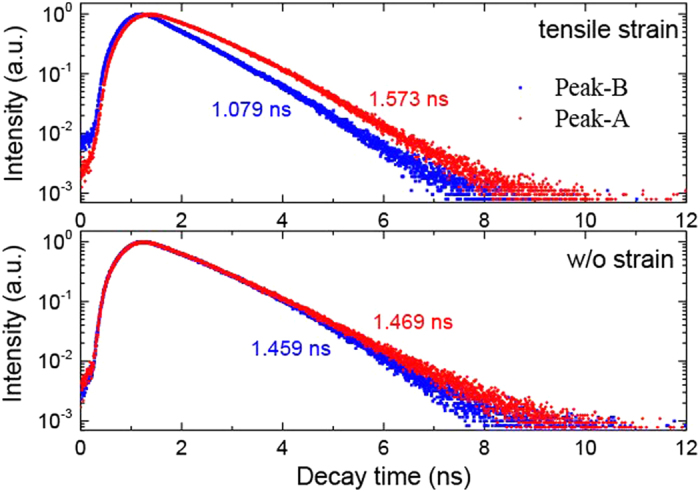
Time-resolved PL. The time-resolved PL of A- and B-exciton measured at crest part of buckled CdS NR where the tensile strain dominated (top panel) and at flat part of buckled CdS NR where strain effect can be ignored (bottom panel).

Strain modulates not just the exciton energies but also the dynamics of exciton emission^22^. Figure 3 (bottom panel) shows the time resolved spectra of A- and B-exciton obtained from the flat part of NR, where the tensile strain effect can be ignored. Fitted by single exponential function, the lifetime of A- and B-exciton was obtained as 1.469 ns and 1.459 ns, respectively. The discrepancy between them is within the time resolution of TCSPS (~50 ps). However, the same measurements at the crest part of buckled NR, where a large tensile strain is applied, show a large lifetime variation for A- and B-exciton (as shown in the top panel). The lifetime of B-exciton becomes much shorter (~1.079 ns), while for A-exciton, it becomes a little longer (~1.573 ns). The difference between them is around 500 ps, which is clearly visible in the time-resolved spectra. This difference can be explained by the enhanced coupling of exciton with LO phonon due to larger energy gap between A- and B-exction. In unstrained CdS NR, the energy gap between A- and B-exciton is around 20 meV, which is much smaller than the LO phonon (~38 meV), this suppressed the decay channel of B-exciton to lower energy via transferring energy to LO phonon^59^. But the case is totally changed when the tensile strain induced a relatively large energy gap (>50 meV) between A and B excitons, as shown in Fig. 2(c). In this case, the B excitons can decay to the lower energy states efficiently via coupling of exciton and LO phonon. And furthermore, this decay channel may be enhanced by the energy broadening of A- and B- excitons under gradient tensile strain which lead to that the transition rule of exciton decay via LO phonon can be easily satisfied. Carrier’s dynamics plays a key role in light efficiency or response rate of optoelectronic devices, and these results show that the strain-induced carrier’s dynamics tuning may be an efficient way to improve the performance of optical devices.

**Figure 4:**
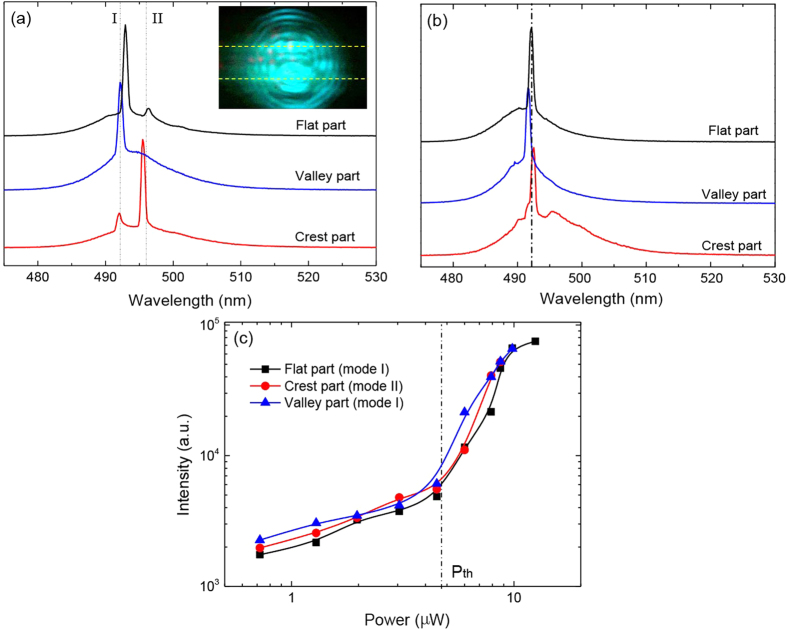
Lasing properties of buckled CdS NRs. (**a**) The lasing spectra measured at flat part (w/o strain), valley part (compressive strain) and crest part (tensile strain) of CdS NRs under the same laser excitation power. Two lasing modes with very little energy shift under different strains are observed. (**b**) Only one lasing mode is observable at gain regime measured on a buckled CdS NR with narrow width (shorter cavity length). (**c**) The intensity of lasing modes (I and II) as a function of excitation power, which shows an almost the same lasing threshold.

NRs served as nanolaser sources have been widely reported in previous works^39^,^41^,^42^, however, the lasing properties of NR under strain have not been investigated. In our samples, the waveguide cavity formed between the two side edges and the optical gains under pulse laser excitation allow us to realize stimulated emission in single CdS NRs. As shown in Fig. 4 (inset), the emission pattern (lasing) from the NR exhibits interference fringes which result from interference between two laser emitting spots from edges^42^. The typical laser spectrum measured from the flat part of NR is shown in Fig. 4(a) (black curve), and two lasing modes (labeled as I and II) are visible in the optical gain region near the bandedge. But when we measure the lasing both at the valley part and the crest part, two obvious features are observed in the spectra (blue and red curves). One is that the competition between two lasing mode is modulated by applying different strain. The compressive strain (valley part) supports the lasing mode I, while the tensile strain (crest part) is favorable for the lasing mode II. This behavior can be well explained by the strain-induced gain shift. Initially, lasing mode I is stronger than lasing mode II (black spectrum), which implies that the highest optical gain located at mode I rather than mode II. At valley part, a compressive strain caused the bandedge blueshifted, and thus the optical gain regime blueshifted accordingly. This suppressed lasing mode II further and made it invisible in blue spectrum. But at crest part, a tensile strain oppositely induced a redshifted bandedge and the optical gain at lasing mode II would be enhanced, leading to stronger lasing mode II than lasing mode I. The other feature is that the energies of lasing modes are quite stable and change very little with strain. As known, the lasing modes are mainly determined by the cavity length and the refractive index of the material. In the planar waveguide cavity formed along the short axis of NR, the width and the thickness of NR is hardly affected because strain is mainly applied along the long-axis of NR. Since CdS has a large dispersion near the bandedge owing to strong coupling between light and excitons, the energy redshift of A-exciton under tensile strain could modulate the refractive index of CdS and thus the cavity modes’ energies. However, under high excitation power, free electron-hole carriers with a large density are generated and all the empty states around the bandedge are completely occupied, in which case, the refractive index is determined by the carrier density (the quasi-Fermi surface in the conduction and valence band) rather than by the energy band^60^. So under the same excitation power, the carrier density in the buckled NR is almost the same regardless of strain, and thus the influence of strain on the refractive index can be ignored. This is why the energies of lasing modes change very little even though a large tensile or compressive strain is applied. In addition, from a point of view of practical applications, the single-mode nanolaser is highly desirable in the future optical digitized communication and signal processing^61^,^62^. Thus we measured a buckled CdS NR with very narrow width (short cavity length, ~1.5 μm) where only one lasing mode is observable in the gain range. Similar to the above multi-mode laser measurements, the lasing spectra measured at the flat part, crest part and valley part of buckled NR are shown in Fig. 4(b). Just as we expected, the lasing mode indeed shifts very little under different strain, which is almost negligible. Furthermore, the emission intensity as a function of excitation power measured at three different strain parts are also presented in Fig. 4(c). Similar to the energies of lasing modes, the threshold of lasing with average power of around 4.7 μW is almost independent of strain. Our results demonstrates that the nanoribbon-based laser sources can retain their optical properties even though a large strain is generated by deformation of devices, this could be very helpful for flexible nanodevices in which the functionalities are not affected by the deformation at all.

## Summary and Conclusions

By using a new but relatively easy method, we have fabricated uniform sinusoidal out-of-plane buckled CdS NRs. This led to a periodic modulation of emission energy with wavelength variation of 14 nm. The contribution of A- and B-exciton to the broadening spectrum under tensile strain were studied experimentally by polarized micro-PL and micro-reflectance, the underlying mechanism is believed to be the fact that A-exciton redshift more than B-exciton under the same tensile strain. Besides that, the dynamics of excitons and the lasing properties under strain were also investigated. Buckled CdS NRs may serve as one of optical elements in flexible optoelectronic devices.

## Additional Information

**How to cite this article**: Wang, Q. *et al*. Emission energy, exciton dynamics and lasing properties of buckled CdS nanoribbons. *Sci. Rep.* **6**, 26607; doi: 10.1038/srep26607 (2016).

## Supplementary Material

Supplementary Information
