# Supplementary material for: Emission energy, exciton dynamics and lasing properties of buckled CdS nanoribbons
Source: Sci Rep. 2016 May 23;6:26607. doi: 10.1038/srep26607 (PMC4876404; doi:10.1038/srep26607)

Supplementary Materials

Emission energy, exciton dynamics and lasing properties of buckled CdS nanoribbons

Qi Wang[1,4], Liaoxin Sun[1*], Jian Lu[3], Ming-Liang Ren[2], Tianning Zhang[1], Yan Huang[1], Xiaohao Zhou[1], Yan Sun[1], Bo Zhang[1], Changqing Chen[4], Xuechu Shen[1], Ritesh Agarwal[2], and Wei Lu[1*]

[1]National Lab for Infrared Physics, Shanghai Institute of Technical Physics, Chinese Academy of Science, Shanghai, 200083, China. [2]Department of Materials Science and Engineering, University of Pennsylvania, Philadelphia, Pennsylvania, 19104, Unite States. [3]Shanghai Advanced Research Institute, Chinese Academy of Science, Shanghai 201210, China. [4]Wuhan National Laboratory for Optoelectronics, Huazhong University of Science and Technology, Wuhan 430074, China. Correspondence and requests for materials should be addressed to L. S. (email: sunlx@mail.sitp.ac.cn), W. L. (email: luwei@mail.sitp.ac.cn).

Fig. S-1

The schematic diagram of experimental configurations for photoluminescence measurement on a single buckling CdS NRs. Lasers: 457 nm continuous laser used for PL and 355 nm pulse laser (20 kHz, 1.1 ns) used for lasing.

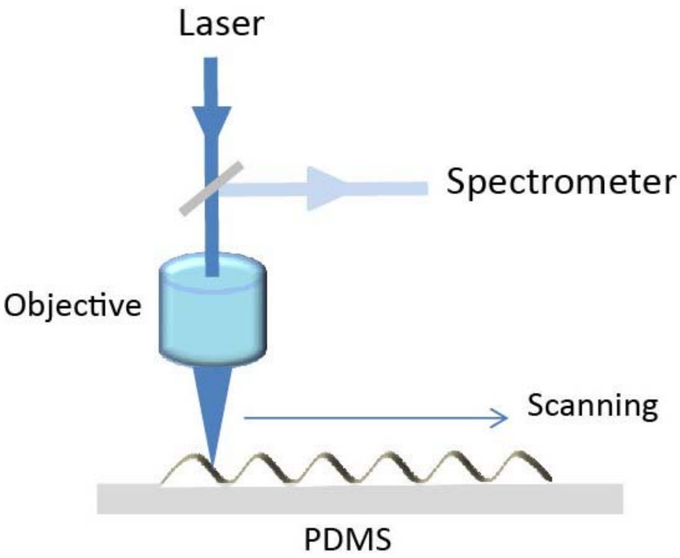

A comparison of PL spectra of nanoribbon with and without a layer of Al_2O_3 at 80 K is shown in fig.S-2. It is obvious that the 5 nm Al_2O_3 has negligible influence on the emission peaks of nanoribbon. The surface passivation can change the dielectric environment of CdS nanoribbons, and this has been well demonstrated in the Ref [1], where a layer of PMMA was coated on the surface of CdS nanoribbons, and a visible redshift was observed for 31 nm thick nanoribbon at room temperature, which is ascribed to the decrease of surface states. But for low temperature PL measurement, the surface passivation doesn't work anymore because the freezing-out of donors leads to a much weaker depletion field. So in our case, the liquid nitrogen PL measurement and large thickness nanoribbons (>200 nm) may be reasons for the negligible PL changing by a layer of Al_2O_3.

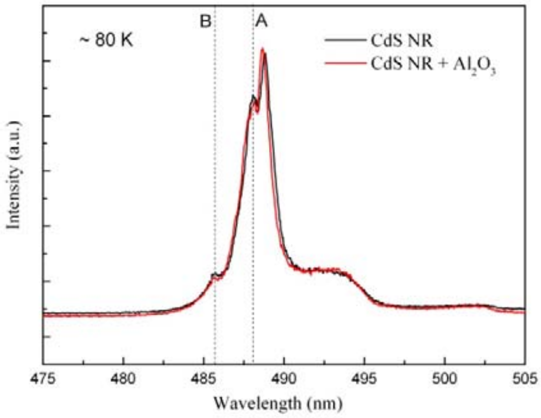

Fig. S-2 PL spectra of a nanoribbon with and without a layer of Al_2O_3.

[1] D. Li, J. Zhang, and Q. Xiong, Surface Depletion Induced Quantum Confinement in CdS Nanobelts, ACS Nano. 6, 5283 (2012).

Fig. S-3

The corresponding waterfall plots of the PL mapping in Figure2 (a). The maximum redshifted emission peaks (~ 503.5 nm) and the emission peaks (~ 489.5 nm) correspond exactly to the crest and the valley of the buckled NR, respectively.

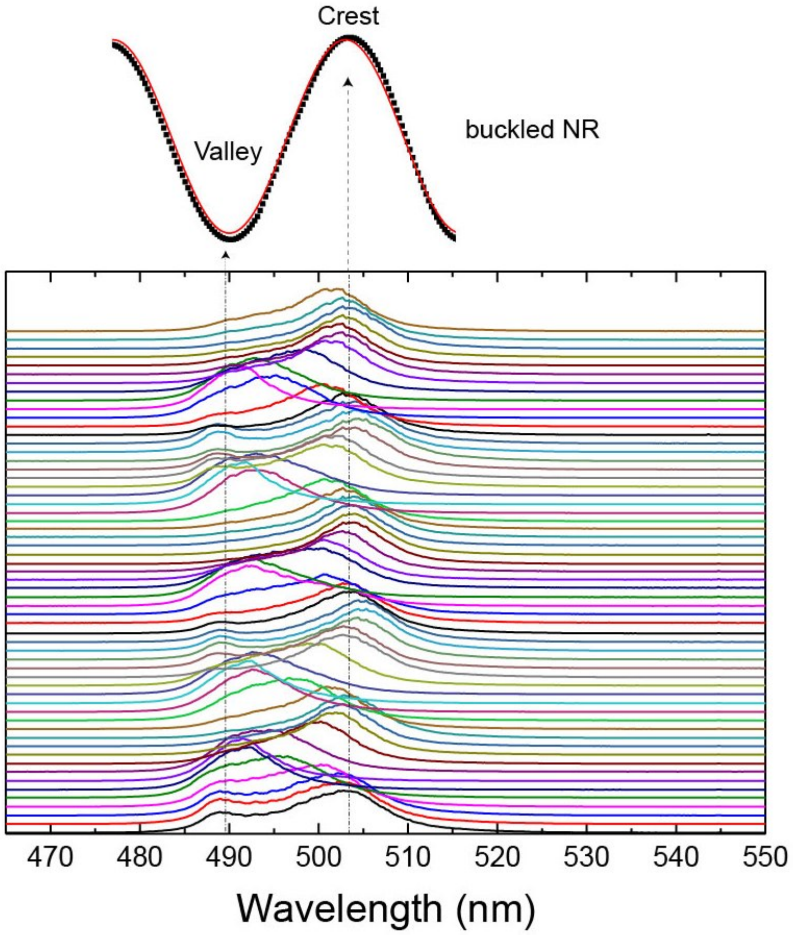

Supplement: Supplementary Information [file srep26607-s1.pdf]
